# Bivalent chromatin states and epigenetic priming in cotton: the role of H3K4me3 and H3K27me3 in cold stress memory and resilience

**DOI:** 10.1186/s13072-026-00680-3

**Published:** 2026-07-01

**Authors:** Mayamiko Masangano, Ahmed Nasre, Ferehewoit Deressegn Lakew, Yohannes Gelaye, Yu Gao, Yuzhi Zhang, Gongye Cheng, Usama Arshad, Lin Ziwei, Li Yang, Meng Kang, Yu Liang, Xiaoyu Cao, Xuehong Song, Chengjun Miao, Bei Liubei, Yan Shihong, Xiaoyu Pei, Xiang Ren, Kunlun He, Abdou Mahaman Mahamadou, Nooney Chidwala, Songjuan Tan, Xingxing Wang, Zhenyu Wang, Xiongfeng Ma

**Affiliations:** 1 State Key Laboratory of Cotton Bio-breeding and Integrated Utilization, Institute of Cotton Research, Chinese Academy of Agricultural Sciences, Anyang, 455000 China; 2 College of Agriculture and Natural Resources, Assosa University, Assosa, Ethiopia; 3 College of Agriculture and Natural Resources, Debre Markos University, Amhara, Ethiopia; 4 Faculty of Tropical Agriculture and Forestry, Hainan University, Haikou, 570228 Hainan China; 5 WeForest asbl/vzw, Cantersteen 47, Brussels, 1000 Belgium

**Keywords:** Cotton, Cold stress, Epigenetic priming, Bivalent chromatin, Stress memory

## Abstract

Cold stress is a significant challenge to cotton (*Gossypium hirsutum L.*) production during seed emergence and early seedling establishment, as cotton is native to tropical and subtropical environments. Low temperatures during these sensitive stages impair photosynthetic efficiency, damage cellular structures, and reduce yield. Although cotton responses to cold stress have been extensively investigated at physiological, molecular, and transcriptional levels, increasing evidence suggests that transient gene expression changes alone are insufficient to explain sustained stress performance. This review synthesizes current knowledge on cotton cold-stress responses, emphasizing the regulatory roles of key histone modifications histone H3 lysine 4 trimethylation (H3K4me3) and histone H3 lysine 27 trimethylation (H3K27me3) in transcriptional control and within-generation (somatic) epigenetic priming. Although cotton cold epigenome profiling is beginning to emerge, cotton-specific, time-resolved chromatin datasets that span chilling, recovery, and recurrent chilling (stress-recovery-re-stress) remain limited; therefore, several mechanistic inferences necessarily rely on indirect evidence from cotton studies under other conditions and on well-characterized model plant systems. H3K27me3 is implicated in Polycomb-mediated gene silencing and may regulate gene reactivation during cold stress and recovery, whereas H3K4me3 is proposed to support rapid induction of cold-responsive genes. Bivalent chromatin domains containing H3K4me3 and H3K27me3 may maintain stress-related genes in a poised transcriptional state, enabling swift activation while preserving developmental regulation. We highlight key knowledge gaps and experimental priorities for establishing cotton-specific chromatin mechanisms of cold memory and for translating these insights into epigenome-assisted breeding and biotechnological strategies to develop cotton varieties with improved and stable cold resilience.

## Introduction

Cotton (Gossypium spp.) is a globally significant natural fiber crop, yet it is sensitive to low-temperature stress. The genus evolved primarily under tropical and subtropical conditions and is therefore susceptible to freezing (< 0 °C) and chilling (0–15 °C) events that are common in temperate growing regions. Cold stress affects germination and early seedling development, reduces stand establishment, and causes substantial yield losses [1–3]. Increasing climate variability and abrupt temperature shifts further amplify this constraint, making low temperature stress a persistent challenge to sustainable cotton production and motivating a deeper understanding of freezing tolerance and chilling resiliency mechanisms [4, 5]. Among abiotic stresses, cold destabilizes membrane fluidity, disrupts protein complexes, severely inhibits photosynthetic activity, perturbs plant water status and enzyme activity, and promotes membrane damage and photoinhibition in cotton [6–8]. Cold-tolerant cultivars such as Zhongmian 36 (ZM36) exhibit marked reductions in transpiration rate, stomatal conductance, net photosynthetic rate, and PSII efficiency, accompanied by elevated intercellular CO_2_ and increased Fo/Fm ratios, consistent with structural injury to the photosynthetic apparatus under cold stress [9].

At the molecular level, low temperature activates complex regulatory networks involving transcriptional reprogramming, calcium signaling, reactive oxygen species (ROS) signaling, and mitogen-activated protein kinase (MAPK) cascades [10, 11]. A central module is the ICE-CBF-COR pathway, in which C-repeat binding factor (CBF) transcription factors bind dehydration-responsive elements in promoters of cold-responsive genes and induce protective outputs such as late embryogenesis abundant (LEA) proteins [12, 13]. Additional transcription factor families, including APETALA2/ethylene-responsive factor (AP2/ERF), NAC, basic helix-loop-helix (bHLH), MYB, and WRKY, also shape downstream cold signaling [14–16]. Transcriptomic analyses further implicate regulators such as GhPLATZ (plant AT-rich sequence and zinc-binding protein), GhUVR8 (UV RESISTANCE LOCUS 8), GhNFYA1 (NUCLEAR FACTOR Y, subunit A1), and GhNHL69 (NDR1/HIN1-like 69) in cotton cold tolerance [17].

Plants can store information from past stress exposure and respond more effectively to subsequent events through epigenetic, rather than genetic, change [18]. In this review, we use epigenetic priming to denote a within-generation (somatic) state of enhanced responsiveness established after a prior, often mild, cold episode and manifested upon re-exposure within the same plant life cycle. We distinguish immediate transcriptional readiness (minutes to hours), somatic epigenetic priming or cold stress memory (days to weeks), and inter- or transgenerational epigenetic inheritance, which is conceptually distinct from priming and remains insufficiently resolved for cold responses in cotton [19, 20]. Chromatin-based regulation in this context includes histone modifications, chromatin remodeling, DNA methylation, and non-coding RNAs. H3K27me3, often deposited by Polycomb repressive complex 2 (PRC2), is associated with stable repression, whereas H3K4me3 is linked to transcriptional activation [21, 22]. These signals can be redistributed dynamically and in a locus-specific manner in response to cold stress and recovery, forming a chromatin-based memory system, while bivalent chromatin states may keep cold-responsive genes poised for rapid inducibility under developmental constraint [20, 23, 24]. Because upland cotton (*Gossypium hirsutum* L.) is an allotetraploid species, its genome contains A-subgenome and D-subgenome homeologs derived from A-genome- and D-genome-related diploid progenitor lineages. In this review, the terms A and D subgenomes refer to these two homeologous genomic compartments, which may differ in chromatin marking, gene expression, and stress responsiveness. Genome-wide analyses further indicate that such chromatin patterns can differ between the A and D subgenomes [22, 25].

We propose that within-generation cold stress memory in cotton may be associated with coordinated, locus-specific reconfiguration of H3K4me3 and PRC2-linked H3K27me3 at key cold-responsive regulatory genes, such that recovery after an initial cold episode leaves a partially reset or poised chromatin state that supports faster transcriptional re-induction upon recurring chilling [18–20, 22]. Because cotton is an allotetraploid, we further hypothesize that A- and D-subgenome homeologs can differ in the magnitude and persistence of this reconfiguration, contributing to subgenome-biased cold responsiveness observed in cotton epigenomic studies [22, 25]. This model yields three testable expectations: loci showing transcriptional memory after cold priming will retain elevated H3K4me3 and/or show delayed re-establishment of H3K27me3 during recovery; bivalent or bivalency-like H3K4me3-H3K27me3 configurations will be enriched at regulatory nodes that must remain developmentally constrained yet rapidly inducible under cold; and memory-associated chromatin signatures will show homeolog and subgenome bias consistent with cotton epigenomic asymmetry [18, 22, 25]. Despite advances in cotton cold-stress physiology and transcriptomics, the chromatin mechanisms that underlie within-generation cold stress memory remain incompletely resolved; accordingly, the following sections summarize cotton physiological and transcriptional responses to cold stress, review cotton histone landscapes, and evaluate H3K4me3 dynamics, H3K27me3/Polycomb regulation, and bivalent chromatin states as candidate mechanisms relevant to developing cold-resilient cotton varieties [26].

## Cold stress in cotton: physiological and molecular responses

Cold stress disrupts membrane fluidity, destabilizes protein complexes, and suppresses photosynthesis in cotton seedlings, thereby reducing vigor during germination and early establishment. These effects translate into poor emergence, slower early growth, and substantial yield penalties when chilling occurs during the most temperature-sensitive developmental window. Cold exposure also induces dehydration, depresses enzyme activity, damages membranes, and lowers photosynthetic efficiency [27–29]. Together, these traits are consistent with photoinhibition and structural injury to the photosynthetic apparatus.

**Fig. 1:**
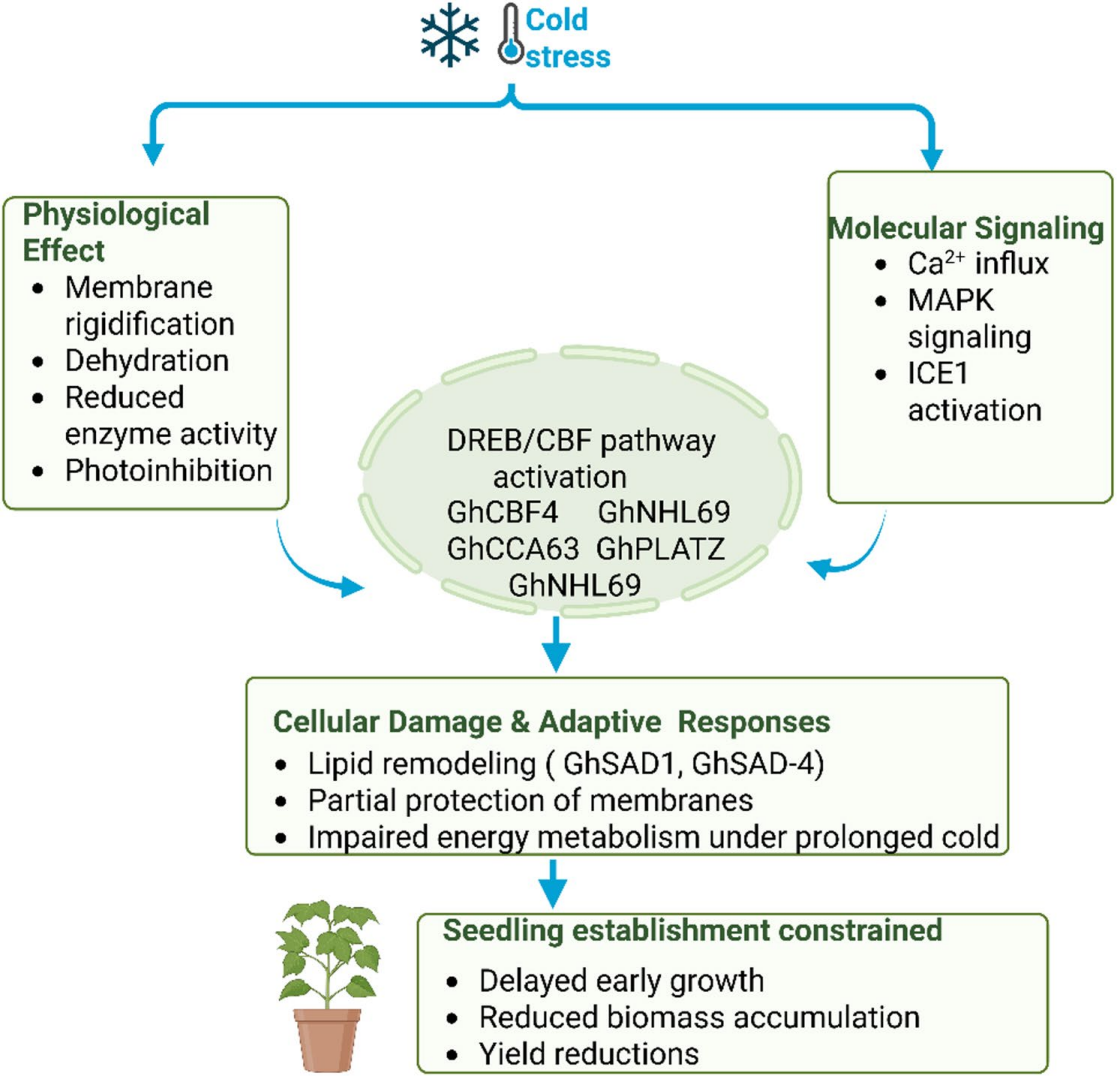
Molecular and physiological responses of cotton to cold stress. The scheme links early signaling events, including calcium (Ca²⁺) influx, reactive oxygen species (ROS) accumulation, and mitogen-activated protein kinase (MAPK) activation, to downstream transcriptional regulation mediated by the dehydration-responsive element-binding (DREB)/C-repeat binding factor (CBF)-cold-responsive (COR) pathway. These molecular processes lead to physiological outcomes such as membrane rigidification, photoinhibition, dehydration, reduced photosynthetic efficiency, and impaired growth

The molecular responses that occur in response to cold stress in cotton are coordinated: calcium (Ca ^2+^) signaling, reactive oxygen species (ROS) accumulation and mitogen-activated protein kinase (MAPK) cascades (Fig. 1). These early signals lead to transcriptional reprogramming pathways, with the most notable one being the DREB/CBF pathway that controls cold-responsive (COR) genes that deal with membrane stabilization and stress protection [13, 30, 31]. Stress signaling is further coupled with growth regulation by additional family transcription factors such as AP2/ERF, NAC, bHLH, MYB and WRKY [32–34]. Transcriptomic analysis has discovered many differentially expressed photosynthetic and carbon metabolism and stress signaling pathway genes. The important regulators such as GhUVR8 (UV RESISTANCE LOCUS 8), GhPLATZ (plant AT-rich sequence and zinc-binding protein), GhNFYA1 (NUCLEAR Factor Y subunit A1), GhFAD4-1/GhFAD4-2 (fatty acid desaturase 4 homologs), and GhNHL69 (NDR1/HIN1-like 69) are involved in cold tolerance in cotton. Conversely, the expression of genes like GhKCS13, GhCBF4 and GhZAT10 has been found to be altered with respect to cold sensitivity [9].

## Cotton epigenomic context relevant to cold stress

Cotton has growing epigenomic resources that provide a baseline for interpreting stress-induced chromatin regulation, including chromatin accessibility maps for ovule and fiber development and multi-omics epigenetic profiling of developing fibers that reveal stage-specific regulation and subgenome-biased control of gene expression in polyploid cotton [35–38].

Most available cotton epigenomic datasets have been generated in developmental contexts, particularly ovule and fiber development, rather than under cold stress [35–38]. These datasets are still useful because they provide baseline information on chromatin accessibility, DNA methylation, histone modifications, three-dimensional chromatin organization, and subgenome-biased gene regulation in cotton [22, 35–38]. However, they cannot be treated as direct evidence for cold-induced chromatin memory. Their relevance to cold stress is mainly indirect: cotton fiber development involves initiation, elongation, secondary cell wall biosynthesis, and maturation, and these developmental processes are strongly influenced by environmental and physiological conditions [28, 38]. Therefore, tissue-specific and stage-specific cold epigenome profiling remains necessary to connect chromatin dynamics with agronomically relevant cold responses in cotton.

**Table 1:** Chromatin- and small-RNA-associated regulators involved in cotton fiber initiation and their functional roles

Regulatory component	Pathways	Role in fiber initiation	Ref.
DNA Methylation (CG/CHG/CHH)	MET1, CMT3, DRM, CMT2	Chromatin maintenance; CG/CHG enrichment; CHH increase; fiber fate	[38–41]
CHH Methylation Pathway	CMT2–H3K9me2 Axis	CHH increase; heterochromatin formation; RdDM-independent	[39, 40]
Methylation–Histone Crosstalk	DNA methylation ↔ H3K27me3	Chromatin accessibility; promoter repression; fiber identity	[42]
Methylation Inhibition	Zebularine	Fiber protrusion blocked; identity commitment loss	[38]
Histone Deacetylation	GhHDA5 (HDAC)	Transcription balance; fewer fiber initials; ROS accumulation; autophagy	[43]
Histone Variant Incorporation	GhH2A12 (H2A variant)	Nucleosome stability; G2/M transition; fiber initiation	[44]
Histone Modifications & Developmental Control	HDACs, H3K27me3, chromatin remodelers	Developmental transitions; hormone responsiveness; fiber chromatin states	[42]
miR156 → SPL9	miR156 → SPL9	Epidermal identity; fiber fate	[45]
miR160 / miR160-3p.2 → ARF3, GASA	miR160/160-3p.2	Auxin/GA signaling; fiber protrusion	[45]
miR166 → HD-ZIP TFs	miR166	Ovule polarity; fiber identity	[46]
miR167 → LIM genes	miR167	Cytoskeleton organization; fiber initiation	[45]
miR397 → Laccase	miR397	Reduces lignification; fiber cell wall loosening	[45]
miR828 / miR858 → MYB2	miR828/858	MYB-dependent fiber identity programs	[47, 48]
miRNA–Chromatin Interaction	miRNAs → hormone– chromatin pathways	Fine-tune chromatin reprogramming; fiber initiation support	[42]

## Histone modification landscape in cotton: focus on H3K4me3 and H3K27me3

H3K4me3 and H3K27me3 are two intensively studied histone modifications that are commonly associated with transcriptional activation and repression, respectively, and they contribute to developmental regulation and stress-responsive gene control in plants [49–52]. However, these marks function within a broader chromatin regulatory system that also includes DNA methylation, histone acetylation, H3K9me2, chromatin accessibility, histone variants, nucleosome positioning, chromatin remodelers, and non-coding RNA-mediated regulation. Allopolyploid cotton (Gossypium hirsutum), with four chromosome sets, exhibits these histone modifications, which are associated with gene-expression coordination post-polyploidisation, facilitating developmental regulation, subgenome divergence, and evolutionary adaptation [22, 25]. Whereas histone methylation has been thoroughly investigated in model organisms, comprehensive genome-wide chromatin-level analyses of H3K4me3 and H3K27me3 in cotton have only recently emerged via high-resolution ChIP-seq and integrative epigenomic methodologies [22, 52].

Genome-wide profiling reveals the distribution of genomic features in cotton. These H3K4me3 marks are highly concentrated downstream of transcription start sites and often extend into gene bodies, a typical pattern observed in genes that are currently being transcribed and found in all plant genomes [53]. On the other hand, H3K27me3 typically covers larger gene-body areas and is strongly associated with transcriptional repression mediated by PRC2, a well-established process in plants [50–52]. The comparative analyses of the A and D subgenomes of cotton reveal that, although the distribution of H3K27me3 is predominantly conserved, H3K4me3 exhibits significant subgenome-specific variation. Specifically, diminished promoter-proximal enrichment and heightened distal intergenic marking of H3K4me3 are evident in the A subgenome, indicating the development of epigenomic asymmetry after allopolyploidisation [37]. Importantly, most genome-wide profiling studies demonstrate associations between histone-mark distribution and transcriptional status rather than direct causality. Causal inference requires functional perturbation of histone writers, erasers, or readers, combined with time-resolved transcriptomic and chromatin profiling.

The functional ramifications of these histone modification patterns are evident in transcriptional divergence and the organization of regulatory networks. Studies of homeologous gene pairs demonstrate that highly expressed homeologs are consistently enriched for H3K4me3. In contrast, their lowly expressed counterparts preferentially accumulate H3K27me3, thereby reinforcing subfunctionalisation and promoting long-term genome stability in polyploid cotton. Cotton genomes also have wide H3K4me3 domains that cover promoters or whole gene bodies. This trait is linked to critical developmental regulators and biological functions specific to each subgenome [22, 25]. The combination of histone modification profiles with chromatin accessibility, three-dimensional genome architecture, and transcription factor network analyses shows that genes marked by H3K4me3 and H3K27me3 are part of coordinated regulatory modules that control the growth of fibers and ovules [35]. Together, these genome-wide profiles indicate that H3K4me3 and H3K27me3 are associated with transcriptional divergence, subgenome-biased chromatin patterning, and developmental regulatory modules in cotton. However, these data primarily support correlations between histone-mark distribution and transcriptional or developmental states, rather than demonstrating direct causality; functional perturbation of histone writers, erasers, or readers will be required to determine whether these marks actively establish the observed regulatory outcomes [22, 35, 36].

## Dynamics of H3K4me3 in cotton under cold stress

A major limitation in evaluating H3K4me3-based cold memory mechanisms in cotton is that cotton-specific, genome-wide H3K4me3 profiling explicitly designed to capture priming, recovery, and re-stress (stress-recovery-re-stress) remains scarce. A recent study profiled genome-wide H3K4me3 and H3K9ac in upland cotton seedlings under acute cold stress using CUT&Tag and reported that changes in these marks correlate with cold stress-responsive gene activation [54]. Integrated multi-omics analyses that include chromatin accessibility (ATAC-seq) have likewise begun to map cold-responsive regulatory networks in cotton [34]. However, comparable time-resolved datasets spanning recovery and re-stress the critical window for testing persistence of H3K4me3 at memory loci are not yet available. Accordingly, the evidence summarized in this section is drawn from (i) emerging cotton cold epigenomic datasets focused on acute stress, (ii) cotton epigenomic studies conducted under non-cold conditions or other abiotic stresses, and (iii) cold-induced H3K4me3 dynamics described in well-characterized model plants. In Arabidopsis, cold exposure triggers rapid, gene-specific redistribution of H3K4me3 that accompanies the activation of cold-responsive genes during acclimation [23]. More broadly, H3K4me3 is frequently associated with stress-induced transcriptional activation across abiotic contexts, including dehydration, heat, and hormone signaling [19], supporting the plausibility of a similar regulatory role during chilling in cotton.

**Fig. 2:**
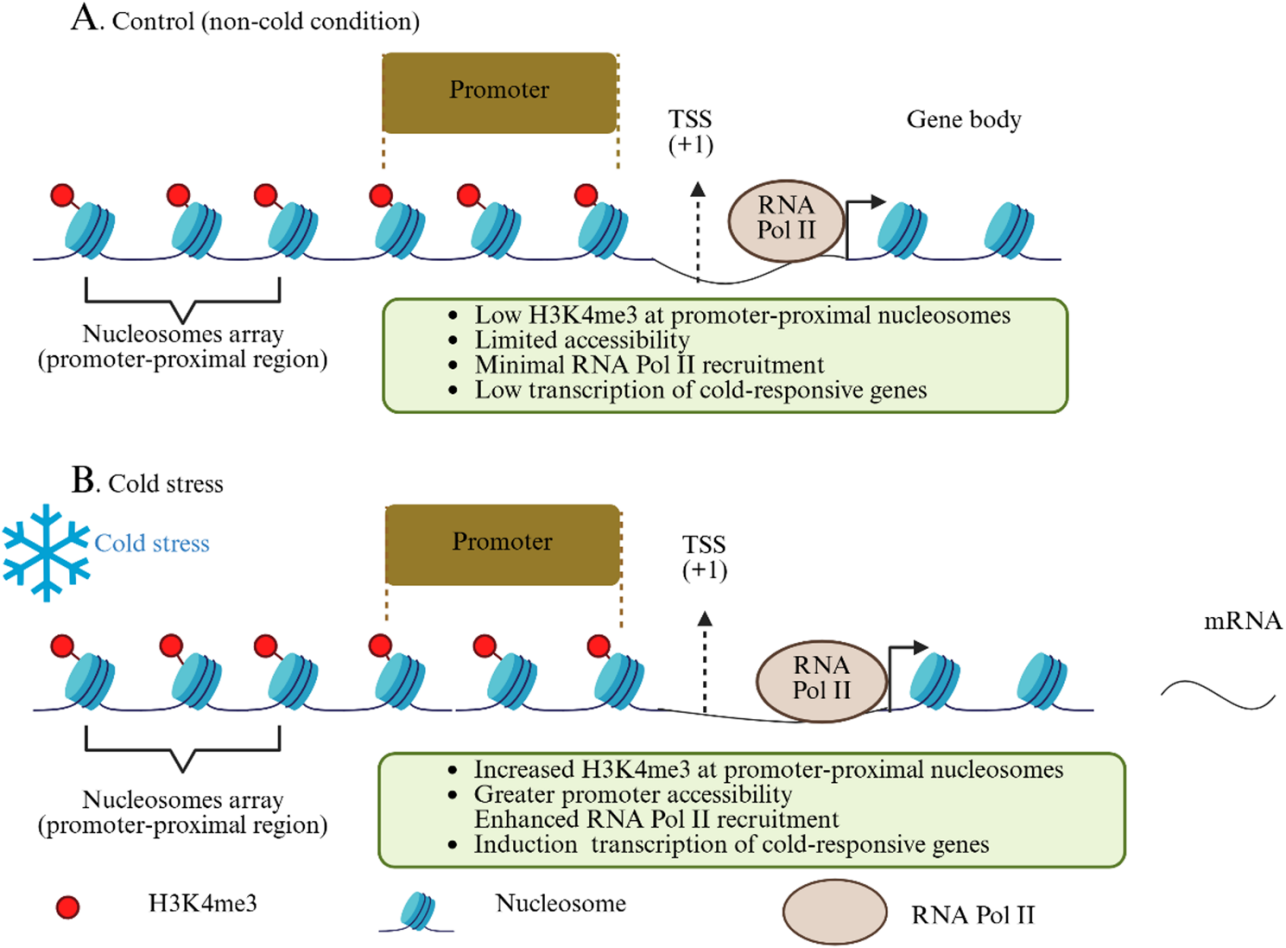
Proposed role of H3K4me3 in transcriptional activation during cotton cold stress. Cold exposure may increase H3K4me3 enrichment on histone H3 tails at promoter-proximal nucleosomes of cold-responsive genes. This activation-associated chromatin state is proposed to increase promoter accessibility, facilitate recruitment of transcriptional machinery near the transcription start site, and support the induction of genes involved in membrane protection, osmotic adjustment, ROS control, and cold tolerance. The model remains hypothetical for cotton cold memory and requires validation using time-resolved chromatin profiling

Cold exposure also drives extensive transcriptional reprogramming in cotton over time, including activation of the ICE–CBF–COR signaling cascade, ROS detoxification, and hormone-regulated pathways [55]. Notably, cotton dehydration-stress memory studies report that subsets of stress-inducible genes retain elevated H3K4me3 after recovery, enabling accelerated reactivation during subsequent stress events [56]. This provides a precedent that H3K4me3 retention can participate in within-generation priming in cotton; however, whether recurrent chilling elicits analogous H3K4me3 dynamics remains untested. Therefore, Fig. 2 is presented as a working hypothesis for cotton that requires direct validation using cold stress-recovery-re-stress chromatin profiling.

Histone demethylases, particularly Jumonji C (JMJ) family proteins, regulate H3K4me3 states by catalyzing removal of H3K4 methylation. Cotton encodes numerous JMJ genes, and stress-responsive expression of subsets of these genes suggests that cold tolerance may depend on balancing H3K4me3 deposition and removal to avoid prolonged activation of energetically costly pathways [57]. Coordinated changes across epigenetic layers, such as promoter DNA demethylation together with activation-associated chromatin marking, have also been linked to transcriptional activation of stress-responsive genes [58]. Available cotton chromatin maps further suggest that subgenome asymmetry is unlikely to resolve into a simple A-subgenome versus D-subgenome hierarchy for cold resilience. Instead, H3K4me3 tends to show stronger subgenome-biased patterning, while the anti-correlation between H3K27me3 and transcription is more pronounced in the A subgenome, implying locus- and homeolog-specific partitioning rather than a uniformly more resilient subgenome [22, 37]. Whether chilling itself reconfigures H3K4me3 in a homeolog-specific manner, and whether such reconfiguration contributes to priming and re-induction kinetics, remains unknown. Consequently, claims about H3K4me3-driven cold memory in cotton should be treated as hypotheses pending cotton-specific cold epigenomic datasets. Knowledge gap and experimental priorities. Definitive tests will require cotton-specific, genome-wide profiling of H3K4me3 during chilling, recovery, and re-stress (example, ChIP-seq or low-input approaches such as CUT&Tag/CUT&RUN), integrated with RNA-seq and chromatin accessibility assays, and analyzed with homeolog-aware mapping across contrasting genotypes and developmental stages. Building on emerging acute-cold datasets in cotton [34] and established low-input chromatin profiling protocols in cotton [59], such stress-recovery-re-stress designs are needed to determine whether H3K4me3 persistence during recovery is a reproducible feature of cotton cold priming.

## H3K27me3-mediated repression and its role in cold response

H3K27me3 is a key repressive chromatin mark that keeps cold-responsive genes inactive under favorable growth conditions. In plants, this mark is deposited by Polycomb Repressive Complex 2 (PRC2), whose core components include CURLY LEAF (CLF), SWINGER (SWN), MEDEA (MEA), FERTILIZATION-INDEPENDENT ENDOSPERM (FIE), and EMBRYONIC FLOWER 2 (EMF2) [60]. PRC1-like readers such as LIKE HETEROCHROMATIN PROTEIN 1 (LHP1) reinforce repression by promoting chromatin compaction and restricting transcriptional access. This basal repression is important because inappropriate expression of cold-effector genes under warm conditions would divert energy from growth [61].

**Fig. 3:**
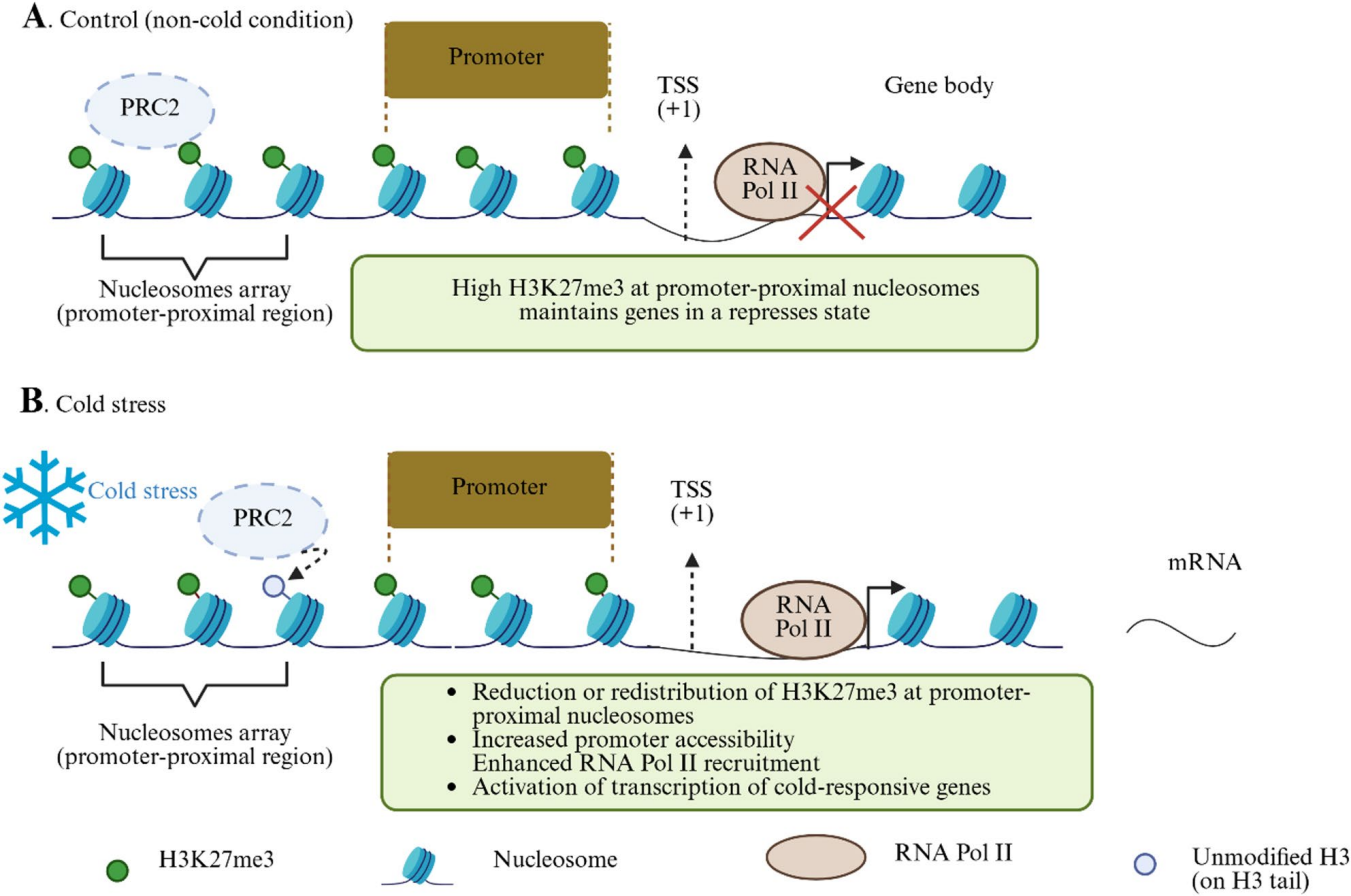
Proposed role of H3K27me3-mediated repression during cotton cold stress. Under non-stress conditions, PRC2-associated H3K27me3 marks on histone H3 tails may maintain selected cold-responsive genes in a repressed or low-expression state. During cold exposure, selective reduction or redistribution of H3K27me3 at promoter-proximal nucleosomes may permit transcriptional activation, whereas repression may be maintained at genes not required for cold adaptation. This model is based mainly on evidence from model plants and remains to be directly tested in cotton

Upon cold exposure, chromatin state can change rapidly and in a locus-specific manner, including selective decreases in H3K27me3 at key cold-responsive loci. In Arabidopsis, H3K27me3 levels decline within hours at promoters of genes such as COR15A, KIN1, and GOLS3, which encode functions linked to membrane stabilization, osmoprotection, and cryotolerance [62]. Importantly, H3K27me3 loss does not necessarily coincide with H3K4me3 gain, arguing against a simple one-to-one antagonistic exchange. Instead, current data support a more specialized and gene-dependent role for H3K27me3 remodeling during cold responses [63]. This proposed H3K27me3-mediated repression model is summarized in Fig. 3.

Some Jumonji C family demethylases contribute to H3K27me3 removal during cold responses. REF6 provides a mechanistic framework by recognizing CTCTGYTY motifs and recruiting chromatin remodelers such as BRAHMA (BRM) to activate stress-responsive genes, whereas ELF6 appears important for maintaining cold-induced H3K27me3 depletion [61, 64]. In Arabidopsis, impaired ELF6 function compromises reactivation of some COR genes during repeated cold exposure, supporting the idea that incomplete re-establishment of H3K27me3 during recovery can influence later responsiveness. For cotton, however, this remains an extrapolative model rather than a demonstrated mechanism. We thus consider H3K27me3 remodeling as a reasonable candidate to cold stress response in cotton but its involvement in within-generation memory is yet to be experimentally confirmed.

## Bivalent chromatin states in cotton cold-stress responses

Bivalent chromatin refers to genomic regions, usually promoter-proximal loci, that show enrichment of both the activation-associated mark H3K4me3 and the repression-associated mark H3K27me3. This chromatin state was first described at key developmental genes in mammalian embryonic stem cells and has also been reported as bivalency-like chromatin in plant systems, including Arabidopsis chromatin-state maps [21, 53, 65]. In principle, this configuration may keep genes in a poised state, allowing rapid activation under environmental cues while maintaining repression under normal growth conditions [21, 66, 67]. However, its interpretation requires caution because co-enrichment detected from bulk ChIP-seq or CUT&Tag data does not necessarily prove that both marks occupy the same nucleosome, allele, or cell type. Apparent co-marking may also reflect population-level averaging, asymmetric nucleosome marking, neighboring-nucleosome configurations, or tissue/cell-type heterogeneity [53, 68, 69].

For cotton cold-stress responses, bivalent chromatin should currently be treated as a testable hypothesis rather than a confirmed mechanism. Existing evidence supports the relevance of H3K4me3 and H3K27me3 in cotton chromatin regulation, including their roles in gene subfunctionalization and subgenome-related chromatin patterning in allotetraploid cotton [22, 25]. Studies in other plant species further suggest that cold-responsive genes may display dynamic combinations of activating and repressive marks. For example, cold stress was associated with H3K4me3 and H3K27me3 dynamics in Arabidopsis and with enhanced chromatin accessibility together with bivalent histone modifications in potato [23, 24, 26]. However, cotton-specific cold datasets capable of distinguishing true co-occupancy from cell-type heterogeneity, homeolog-specific marking, or transient chromatin switching remain limited.

**Fig. 4:**
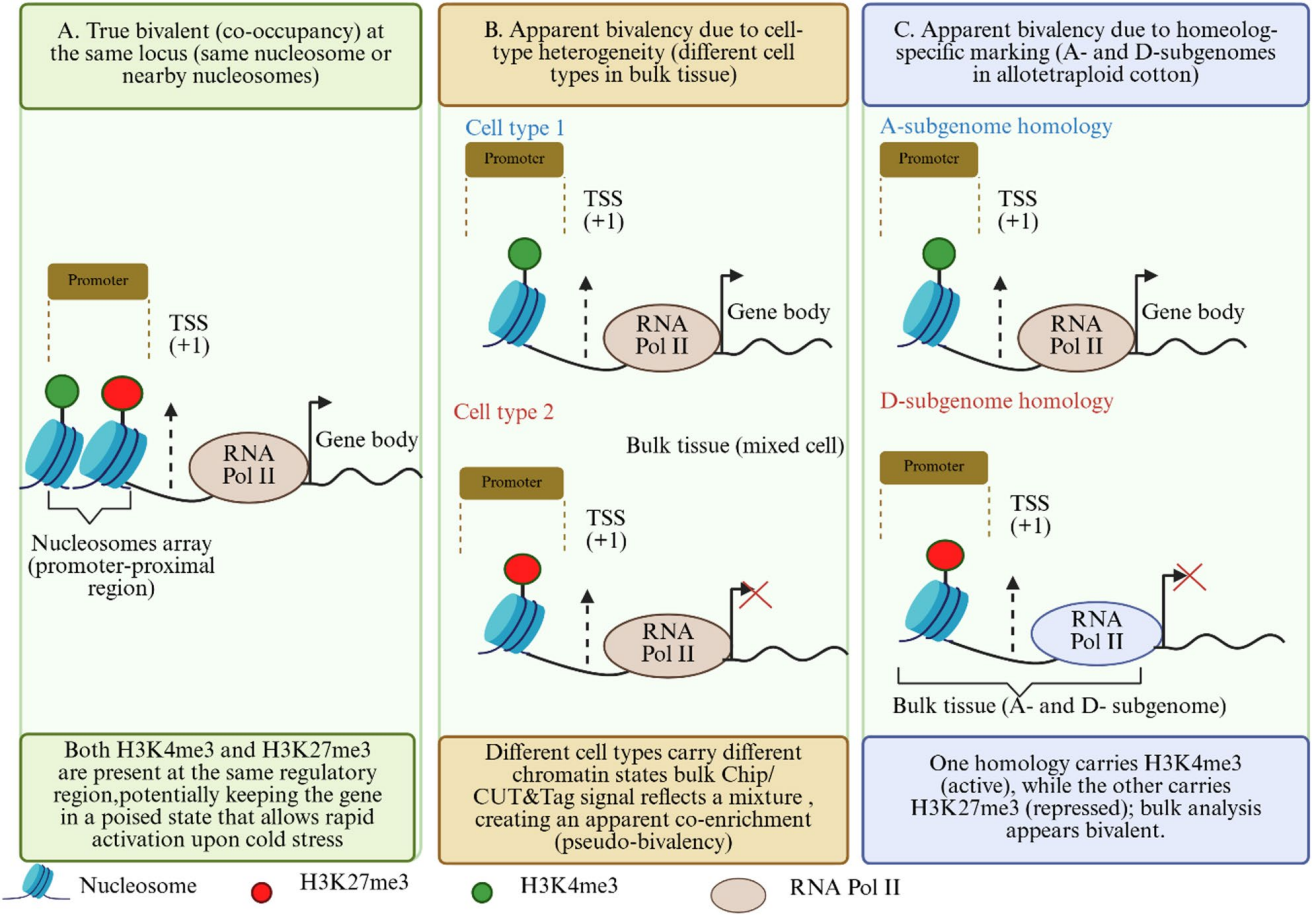
Alternative interpretations of bivalency-like H3K4me3–H3K27me3 patterns in cotton. Co-enrichment of H3K4me3 and H3K27me3 in bulk chromatin profiles may reflect true co-occupancy at the same regulatory locus, cell-type heterogeneity within sampled tissues, or homeolog-specific marking between A- and D-subgenome copies in allotetraploid cotton. Therefore, bivalency-like patterns in cotton cold-stress responses should be interpreted cautiously until validated by sequential ChIP/CUT&Tag, cell-type-resolved profiling, or homeolog-aware chromatin analysis

A conservative model is that selected cold-responsive regulatory genes in cotton may retain activation-associated H3K4me3 while undergoing partial or delayed resetting of H3K27me3 during recovery. Such a state could support faster transcriptional re-induction during recurrent chilling without causing permanent activation of stress pathways. This model is consistent with broader plant stress-memory concepts and with cotton evidence showing stress-associated H3K4me3 retention during dehydration recovery, but it has not yet been directly demonstrated for cotton cold stress [20, 26, 70]. Testing this model will require sequential ChIP/CUT&Tag, multi-mark profiling, cell- or tissue-specific chromatin assays, and homeolog-aware mapping across cold exposure, recovery, and re-stress. These alternative interpretations of bivalency-like H3K4me3–H3K27me3 patterns in cotton are summarized in Fig. 4.

## Epigenetic priming in cotton: concepts and evidence

In this review, epigenetic priming in cotton refers specifically to within-generation enhancement of responsiveness after prior stress exposure, not to proven meiotic inheritance. Direct evidence in cotton is strongest for somatic priming under other abiotic stresses, whereas cold-specific priming remains inferred largely from transcriptomic behavior and from analogies to better resolved systems [56]. This distinction matters because immediate signaling, somatic memory through recovery, and true transgenerational inheritance operate on different timescales and should not be conflated. DNA methylation is one candidate layer in this process. In cotton and other plants, stress-associated changes in promoter or nearby transposable-element methylation can alter transcriptional responsiveness during recovery and subsequent stress exposure. Because RdDM and CHH methylation are especially important for regulating repeat-rich regions in plant genomes, a useful working model is that methylation changes near cold-responsive loci modulate accessibility and interact with histone-based activation or repression rather than functioning independently [58, 70, 71]. Evidence that such methylation states are meiotically inherited after cold in cotton is currently insufficient. Together, these observations support the idea that chromatin-based regulation may be coordinated with RNA-mediated regulation during priming in cotton, although little is known with regard to how it is directly affected by cold stress.

Histone-associated priming is better supported conceptually, but the cotton cold literature remains incomplete. In other abiotic contexts, retention of activation-associated marks such as H3K4me3 or H3K9ac after stress has been linked to faster re-induction of responsive genes during re-exposure. In cotton cold responses, existing datasets support correlations between chromatin activation marks and gene induction, but they do not yet establish persistent memory marks through recovery [56]. Thus, histone-based priming in cotton cold stress should presently be framed as a testable mechanism rather than a confirmed one. Small interfering RNAs (siRNAs), microRNAs (miRNAs), and other small RNAs likely contribute to priming by directing DNA methylation, modulating chromatin state, and fine-tuning transcript abundance at stress-responsive loci. In cotton, small-RNA pathways are well documented in development and in responses to several stresses, but their direct contribution to cold priming and memory remains unresolved. Taken together, the most defensible conclusion is that cotton probably integrates DNA methylation, histone modification, chromatin remodeling, and RNA-mediated regulation during repeated stress, while the relative weight of each layer under cold remains to be determined experimentally [70–72].

## Cold stress memory: proposed chromatin-based mechanisms

Cold stress memory is best viewed as a set of chromatin states that bias future transcriptional responses rather than as a single mark with universal causal power. Among the candidate mechanisms, dynamic regulation of H3K27me3 during stress and recovery is particularly relevant because reduced H3K27me3 at cold-responsive loci can lower the barrier to re-induction during subsequent exposure. In Arabidopsis, loci such as COR15A and GOLS3 exemplify this principle, but equivalent cotton loci have not yet been resolved in comparable time-resolved chromatin datasets [62, 73]. Table 2 therefore summarizes proposed mechanisms from plant systems and indicates where cotton evidence is currently indirect.

A complementary example comes from cucumber, where recovery after cold priming is accompanied by selective H3K27me3 behavior at different classes of genes. Memory-associated genes can reacquire or retain H3K27me3 differently from non-sustainably induced genes, consistent with a role for H3K27me3 in sharpening which transcriptional programs remain responsive after recovery [74]. These findings are informative for cotton, but they should be interpreted as comparative evidence rather than direct proof of the same mechanism in cotton.

**Fig. 5:**
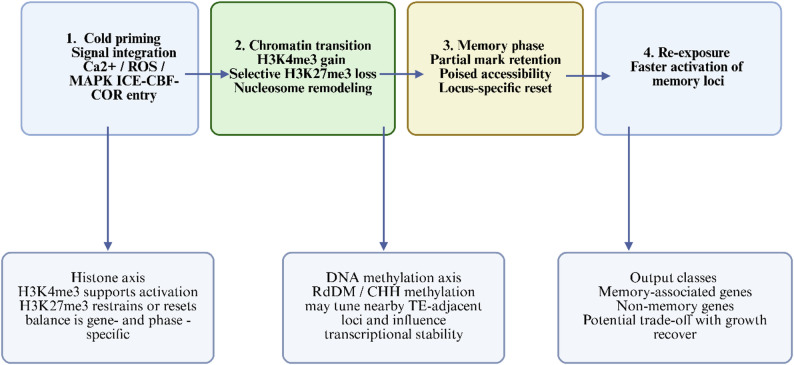
Integrated model of chromatin-based regulation of cold stress responses in cotton. The diagram summarizes the coordinated roles of chromatin modifications, including activating marks such as H3K4me3, repressive marks such as H3K27me3, DNA methylation, and chromatin remodeling, in regulating gene expression during cold stress, recovery, and re-stress. These regulatory layers interact with transcriptional networks to shape stress responsiveness, potentially contributing to within-generation priming while maintaining overall growth regulation

Chromatin remodeling complexes, including SWI/SNF family remodelers, likely act together with histone modifications by repositioning nucleosomes and altering accessibility at responsive loci [20]. A more realistic model in cotton involves coordinated interactions among activating and repressive histone marks, DNA methylation, and chromatin remodeling across priming, recovery, and re-stress. This integrated view better explains how inducibility can be accelerated without globally de-repressing growth programs [20, 26, 58]. This integrated model of chromatin-based regulation during cold stress, recovery, and re-stress is summarized in Fig. 5.

**Table 2:** Chromatin-based mechanisms proposed to underlie cold stress memory in plants

Mechanism	Evidence context/representative loci	Chromatin feature(s)	Proposed role in memory	Key refs.
H3K27me3 repression/remodeling	Arabidopsis cold response and recovery; COR15A, AtGOLS3, other COR-type loci	H3K27me3	Repression at warm temperature; selective depletion or resetting during cold and recovery	[62, 73]
H3K27me3-linked cold memory	Cucumber cold-priming system; memory genes and NSI genes	H3K27me3	Differential resetting distinguishes memory-associated genes from non-sustainably induced genes	[74]
Chromatin remodeling	Stress-responsive loci across plant systems; cold-responsive genes	Remodeling with H3K4me3/H3K27me3 context	Nucleosome repositioning and accessibility changes that support re-induction	[20, 58]
H3K4me3-associated priming	Arabidopsis and other abiotic stress models; cotton candidate loci in the ICE-CBF-COR / COR-like network	H3K4me3, sometimes alongside H3K27me3	Transcriptional priming, partial mark retention, and faster reactivation upon re-stress	[23, 56, 75]
DNA methylation/RdDM context	Cotton epigenomic context and broader plant stress studies; TE-adjacent or repeat-rich regions	CHH methylation, RdDM	May stabilize or reset local regulatory states around stress-responsive loci near repeats	[40, 58, 71]

Representative systems and loci are included to improve interpretability. Evidence is strongest in Arabidopsis and cucumber, whereas several cotton-relevant mechanisms remain candidate models inferred from transcriptomic and epigenomic context rather than from direct cold-stage chromatin profiling

## Knowledge gaps and future priorities

Several major gaps still limit mechanistic interpretation of cotton cold memory. First, we lack cotton-specific time-resolved chromatin datasets that span untreated control, first cold exposure, recovery, and re-stress in the same genotype and tissue. Without this design, transient activation cannot be distinguished from genuine priming. A second gap concerns the role of transposable elements (TEs) and repeat-associated chromatin in cotton cold responses. Because the cotton genome is TE-rich, cold-induced changes in TE expression or nearby CHH methylation could shape accessibility and gene regulation at adjacent cold-responsive loci. Yet the molecular logic by which TE activity, DNA methylation, and histone marks interact under chilling remains poorly defined [46, 58, 71]. A third gap is methodological. Although RNA-seq, ChIP-seq or CUT&Tag, ATAC-seq, and whole-genome bisulfite sequencing have each advanced cotton epigenomics, few studies combine these layers in matched cold experiments across multiple genotypes and developmental stages. Functional validation is also still sparse. Integrating multi-omic profiling with genome editing, reporter assays, and homeolog-aware mapping will be essential for distinguishing correlation from mechanism [46, 56, 58].

A fourth gap concerns non-coding RNAs. Long non-coding RNAs and small RNAs are promising candidates for directing chromatin regulators to specific loci, but the repertoire of cold-responsive RNAs with validated chromatin functions in cotton is largely unknown. Defining these RNA-guided modules is important because they could explain how broadly conserved epigenetic enzymes achieve locus specificity under cold. Finally, the question of inheritance remains open. Somatic priming within a plant life cycle and true intergenerational or transgenerational inheritance are conceptually distinct, and cotton evidence for the latter under cold is currently inadequate. Future work should test inheritance explicitly across generations rather than infer it from within-generation persistence of chromatin states [24, 70].

## Conclusion

Cold stress severely constrains cotton during germination and early seedling establishment, and existing physiological and transcriptomic studies clearly show activation of Ca^2+^ signaling, MAPK cascades, ROS control, and the ICE-CBF-COR network. What remains unresolved is how some responses are rendered more inducible after prior exposure. The most plausible chromatin-level candidates are locus-specific reconfiguration of H3K4me3, H3K27me3, chromatin accessibility, and DNA methylation during stress and recovery. In cotton, however, direct evidence is still strongest for acute cold-associated chromatin changes, not for fully resolved memory mechanisms. Current data support a model in which homeolog-specific chromatin asymmetry, partial resetting after recovery, and possible bivalency-like states could contribute to within-generation priming, but these ideas remain hypotheses until tested in time-resolved, cotton-specific experiments. The priority now is clear: integrate homeolog-aware chromatin profiling, transcriptomics, and functional perturbation across priming, recovery, and re-stress, then connect validated loci to breeding pipelines. This approach offers a realistic route toward cotton varieties with more stable cold resilience under variable climates.

## Data Availability

Data sharing is not applicable to this article, as no new data were generated.

## Abbreviations

AP2/ERF: Apetala2/ethylene-responsive factor
ARF: Auxin response factor
bHLH: Basic helix–loop–helix
BRM: Brahma chromatin-remodeling ATPase
Ca²⁺: Calcium ion
CBF: C-repeat binding factor
CG: Cytosine–guanine sequence context
CHG: Cytosine–any base–guanine sequence context
CHH: Cytosine–any base–any base sequence context
ChIP-seq: Chromatin immunoprecipitation sequencing
CLF: Curly leaf
CMT: Chromomethylase
CMT2: Chromomethylase 2
CMT3: Chromomethylase 3
CO₂: Carbon dioxide
COR: Cold-responsive
CRISPR–Cas9: Clustered regularly interspaced short palindromic repeats–CRISPR-associated protein 9
CRT: C-repeat
DRE: Dehydration-responsive element
DREB: Dehydration-responsive element-binding protein
DRM: Domains rearranged methyltransferase
ELF6: Early flowering 6
EMF2: Embryonic flower 2
Fo/Fm: Minimal to maximal chlorophyll fluorescence ratio
Fv/Fm: Maximum quantum efficiency of photosystem II
GA: Gibberellic acid
GASA: Gibberellic acid-stimulated Arabidopsis gene
GOLS3: Galactinol synthase 3
H2A: Histone 2 A
H3K4me3: Trimethylation of histone H3 at lysine 4
H3K9ac: Acetylation of histone H3 at lysine 9
H3K9me2: Dimethylation of histone H3 at lysine 9
H3K14ac: Acetylation of histone H3 at lysine 14
H3K27me3: Trimethylation of histone H3 at lysine 27
HATs: Histone acetyltransferases
HDACs: Histone deacetylases
HD-ZIP: Homeodomain-leucine zipper
HSPs: Heat shock proteins
ICE: Inducer of CBF expression
JMJ (JmjC): Jumonji C domain-containing protein
LEA: Late embryogenesis abundant
LHP1: Like heterochromatin protein 1
LIM: Lin-11, Isl-1, and Mec-3 domain protein
lncRNA: Long non-coding RNA
LTR: Long terminal repeat
MAPK: Mitogen-activated protein kinase
MEA: Medea
MET1: Methyltransferase 1
miRNA: microRNA
MYB: Myeloblastosis transcription factor
NAC: NAM, ATAF, and CUC transcription factor family
ncRNA: Non-coding RNA
NSI: Non-sustainably induced genes
OBP1: OBF-binding protein 1
PRC1: Polycomb repressive complex 1
PRC2: Polycomb repressive complex 2
PSII: Photosystem II
RdDM: RNA-directed DNA methylation
REF6: Relative of early flowering 6
RNAi: RNA interference
RNA-seq: RNA sequencing
ROS: Reactive oxygen species
siRNA: Small interfering RNA
sRNA: Small RNA
SPL: Squamosa promoter binding protein-like
SWI/SNF: Switch/Sucrose non-fermentable chromatin-remodeling complex
TE: Transposable element
TF: Transcription factor
TrxG: Trithorax group proteins
WGBS: Whole-genome bisulfite sequencing
WRKY: WRKY transcription factor family
